# A systematic review on gender dysphoria in adolescents and young adults: focus on suicidal and self-harming ideation and behaviours

**DOI:** 10.1186/s13034-023-00654-3

**Published:** 2023-09-21

**Authors:** Elisa Marconi, Laura Monti, Angelica Marfoli, Georgios D. Kotzalidis, Delfina Janiri, Cecilia Cianfriglia, Federica Moriconi, Stefano Costa, Chiara Veredice, Gabriele Sani, Daniela Pia Rosaria Chieffo

**Affiliations:** 1 Clinical Psychology Unit, Fondazione Policlinico Universitario Agostino Gemelli IRCCS, Largo Agostino Gemelli 1, 00168 Rome, Italy; 2 Catholic University of the Sacred Heart—Rome, Largo Francesco Vito 1, 00168 Rome, Italy; 3 NESMOS Department (Neurosciences, Mental Health, and Sensory Organs), University of Rome “La Sapienza”, Via Di Grottarossa1035-1039, 00198 Rome, Italy; 4 Department of Psychiatry, Department of Neuroscience, Head, Neck and Thorax, Fondazione Policlinico Universitario Agostino Gemelli IRCCS, Largo Agostino Gemelli 1, 00168 Rome, Italy; 5 UOSD Operative Unit Psychiatry and Psychotherapy for Adolescents, Azienda USL Di Bologna, Ospedale MaggioreLargo Bartolo Nigrisoli, 2, 40133 Bologna, Italy; 6 Pediatric Neuropsychiatry Unit, Fondazione Policlinico Universitario Agostino Gemelli IRCCS, 00168 Rome, Italy; 7 Institute of Psychiatry, Department of Neuroscience, Catholic University of the Sacred Heart—Rome, Largo Francesco Vito 1, 00168 Rome, Italy; 8 Departement of Life Sciences and Public Health Department, Catholic University of Sacred Heart, 00168 Rome, Italy

**Keywords:** Gender dysphoria, Transgender, Suicide, Suicidal thinking, Suicidal attempts, Non-suicidal self-harm

## Abstract

**Introduction:**

Gender dysphoria (GD) is characterized by the incongruence between one’s experienced and expressed gender and assigned-sex-at-birth; it is associated with clinically significant distress. In recent years, the number of young patients diagnosed with GD has increased considerably. Recent studies reported that GD adolescents present behavioural and emotional problems and internalizing problems. Furthermore, this population shows a prevalence of psychiatric symptoms, like depression and anxiety. Several studies showed high rates of suicidal and non-suicidal self-injurious thoughts and behaviour in GD adolescents. To increase understanding of overall mental health status and potential risks of young people with GD, this systematic review focused on risk of suicide and self-harm gestures.

**Methods:**

We followed the PRISMA 2020 statement, collecting empirical studies from four electronic databases, i.e., PubMed, Scopus, PsycINFO, and Web of Science.

**Results:**

Twenty-one studies on GD and gender nonconforming identity, suicidality, and self-harm in adolescents and young adults met inclusion criteria. Results showed that GD adolescents have more suicidal ideation, life-threatening behaviour, self-injurious thoughts or self-harm than their cisgender peers. Assessment methods were heterogeneous.

**Conclusion:**

A standardised assessment is needed. Understanding the mental health status of transgender young people could help develop and provide effective clinical pathways and interventions.

**Supplementary Information:**

The online version contains supplementary material available at 10.1186/s13034-023-00654-3.

## Introduction

Gender dysphoria (GD) is a condition characterized by a marked incongruence between one’s experienced and expressed gender and the one assigned at birth and is often associated with clinically significant distress or impairment in social, occupational, or other important areas of functioning, especially when reported early [1]. In recent years, the number of young patients diagnosed with GD or and gender-diverse identity—including nonbinary and questioning sexual identities—has considerably increased [2–5]. Current studies document that this population may be exposed to a higher risk of adverse events affecting health status and well-being [6, 7]. This further impacted this vulnerable population, with the most negative consequences for those who experience a gender not congruent with the one they were assigned at birth [8–10]. Indeed, children and adolescents with GD and transgender or transgender and gender nonconforming (TGNC) are described as a psychologically and socially vulnerable population, facing a wide range of physical and mental health concerns that could benefit from early intervention [11–13]. GD during adolescence develops in individuals whose brain is still developing to reach full maturity only some years later, hence the need to dedicate special attention to this population.

As a population perceiving gender minority stress [14], adolescents with GD are likely to lack social acceptance and suffer stigma laid upon them by others [15], but also tend to internalisation [16]. A corollary may be that several studies found adolescents with GD, compared to their age-matched cisgender peers, to show more often behavioural and emotional problems and higher levels of individual distress-generating internalising problems, rather than environment-perturbing externalising problems [17–19]. Consequently, adolescents with GD show a higher prevalence of psychiatric issues, such as depression and anxiety disorders [17, 20, 21], likely due to social stigma.

Adolescents and young adults with GD and gender-diverse identity report higher suicidal thinking, planning, and attempts as well as non-suicidal self-harming thoughts and behaviours (NSSI) than the general population [22–32]. Suicidality is an umbrella term including suicidal ideation, suicidal behaviours, and suicide attempts and plans which are correlated to the desire to die [33]; we will use suicidality sparingly in this paper and focus instead upon the above-mentioned specific terms, when possible. Non-suicidal self-harming behaviours and thoughts, however, refer to self-injurious acts without intending to end one’s own life, but involve self-punishment or negative emotion regulation [34]. In both cases, early age at onset has been identified as an important vulnerability factor, with onset during childhood and adolescence being associated with a poorer prognosis [17], based on different surveys of high-school students [31]. For example, in New Zealand, 20% of students with GD reported attempting suicide in the past 12 months, compared to 4% of all students [35]. Similarly, in the United States, 15% of students with GD reported a suicide attempt requiring medical treatment in the last 12 months, compared to 3% of all students [36–38]. In another American survey, 41% of students with GD reported having attempted suicide during their lifetime, compared to 14% of all students [39]. Moreover, Surace and her colleagues [40] found a mean prevalence of 28.2% for NSSI, 28.0% for suicidal ideation, and 14.8% for suicide attempts in young TGNC clinical populations up to 25 years old.

Besides aspects of GD like body dysmorphic disorder, feeling uncomfortable in one’s own body, and hopelessness about obtaining gender-affirming medical procedures, a possible contribution to elevated suicidal risk and behaviours in the GD population might lie within the social stigma experienced by TGNC adolescents, such as discrimination, prejudice, social stress, and ostracism within the peer group and/or family [41]. Suicidal ideation and self-injurious behaviours generally relate to significant emotional problems, such as depressive and anxiety symptoms, which in turn trigger psychosocial and biological imbalance, could increase the wish to die [42–45], thus adding to the above.

In summary, several studies have shown higher rates of suicidal and non-suicidal self-harming thoughts and behaviours in adolescents and young adults with GD and gender-diverse identity—such as nonbinary and questioning sexual identity—compared to their male and female cisgender peers. However, the evidence heretofore is piecemeal, probably due to social stigma currently associated with GD and the concern of stigmatising individuals suffering from this condition. To better understand the mental state of adolescents and young adults with TGNC, we conducted a systematic review focusing on the risk for suicide and self-harming gestures in the GD population. The aim of this review was to estimate the frequency of suicidal and self-harm behaviour in adolescents and young adults with GD, comparing them with cisgender adolescents where possible.

## Method

We performed a systematic review in compliance with the 2020 PRISMA guidelines for systematic reviews and meta-analyses [46] to increase comprehensiveness and transparency of reporting.

### Information sources and database search

To systematically collect empirical studies on the possible relation between suicidality/self-harming and GD in adolescents and young adults, several keywords were used to search for appropriate publications in four electronic databases, i.e., PubMed, Scopus, PsycINFO, and Web of Science since their inception and no date or language restriction.

Authors conducted the search separately in each database using the following agreed upon search strategy for PubMed and adapting the search for the other databases: (suicid* OR self-injur* OR self-harm* OR self-inflict* OR self-lesion*) AND (gender dysphori* OR transgender) AND (child* OR adolesc* OR "young adult*" OR youth* OR "school age"). Since the terms GD and transgender are used by many people as synonymous, in our searches we used both terms to identify possible eligible articles.

### Eligibility criteria

Inclusion criteria were a study published in a peer-reviewed journal, reporting data on suicide and related behaviours (thinking, planning, and attempts) and/or non-suicidal self-harming thoughts and acts (using methods that reliably obtain the desired result) in adolescent and young adult (14–27 years old) samples with GD/transgender status/gender diverse identity.

Exclusion criteria were studies conducted on children or adult samples and those with mixed populations not providing data for adolescents and young adults separately. Also, opinion papers, such as editorials, letters to the editor, and hypotheses without providing data were excluded, as well as case reports or series, reviews/meta-analyses, animal studies, studies with inadequate/poor methodology and inadequate reporting of data, unfocused, or unrelated to the subject matter. All inter- and intra-database duplicates were removed, as well as abstracts, meeting presentations and studies presenting incomplete data.

Although reviews and meta-analyses were not included, their reference lists were screened to identify additional eligible publications. Eligibility for each study was decided with Delphi rounds among all authors until complete consensus was reached.

### Data extraction

The analysis was conducted by all authors, who applied the eligibility criteria on each database. Each author conducted the selection process separately from others; at a final step, all authors compared their results in Delphi rounds (either in-person or remotely) aimed at obtaining full consensus.

### Data collection and risk of bias assessment

Data collected for each study included country of origin, number of paediatric patients, demographic information (age and biological sex), presence/absence of GD and if present, type of GD, and clinical symptoms focused on self-harm (suicide behaviour, suicidal ideation, suicidal intent and planning, non-suicidal self-harm, and other self-injurious behaviour).

The evaluation of the risk of bias was conducted by a quality index derived from the Qualsyst’ Tool [47]. The quality of selected studies was assessed independently by all investigators and disagreements were resolved by consensus (results of risk-of-bias for all studies in the Additional file 1).

## Results

### Identified studies

**Fig. 1:**
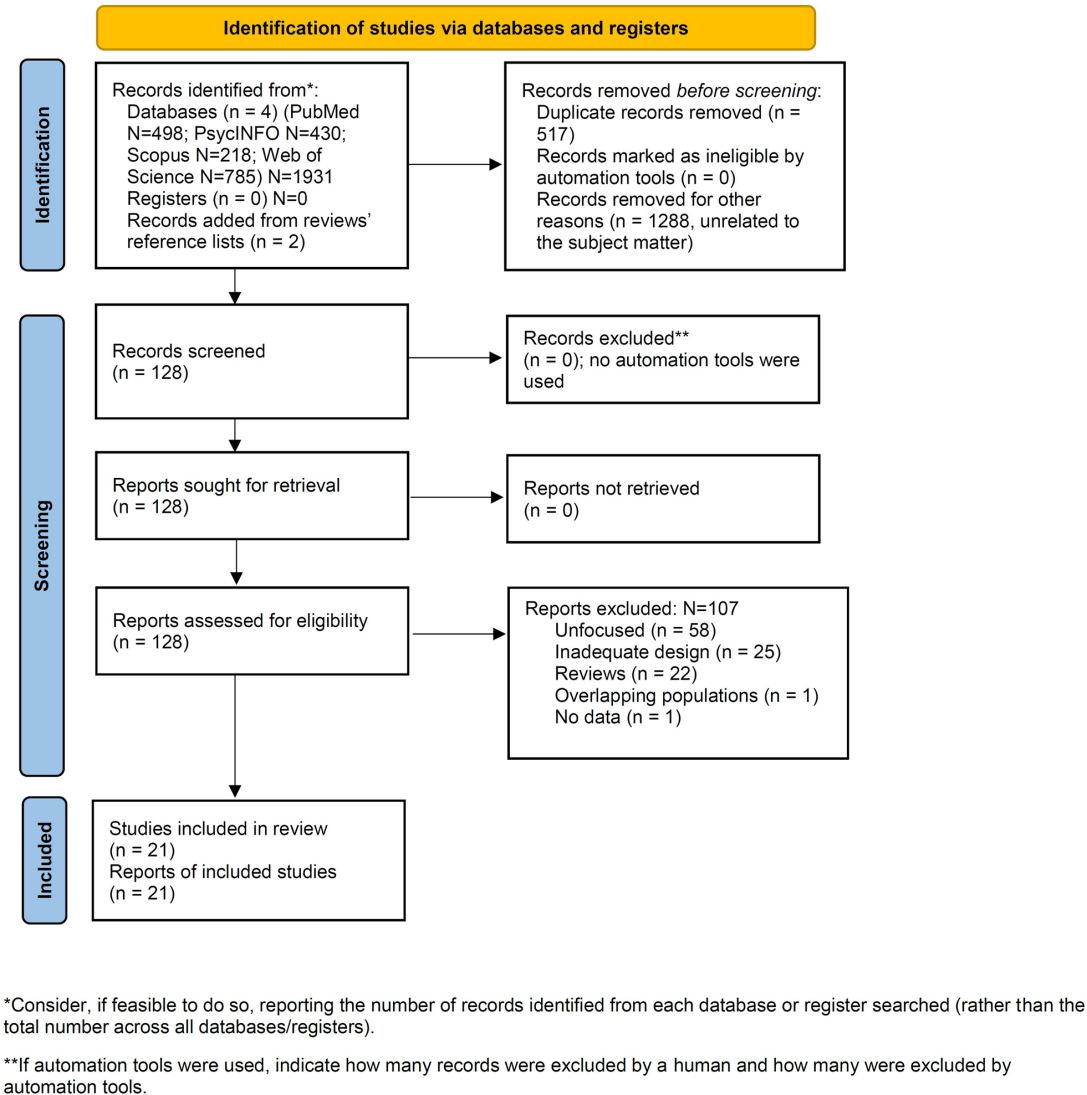
PRISMA2020 flow diagram for new systematic reviews: Search findings and selection with detailed reasons for exclusion

On February 7, 2023, we located 1416 articles (Fig. 1, PRISMA flowchart) [39], of which 128 articles were assessed for eligibility. Of these papers, 107 articles were excluded according to eligibility criteria; 21 dealing with the relationship between GD and gender non-conforming identity, and suicidality and self-harm in adolescents and young adults met inclusion criteria.

For the purpose of this systematic review, we have focused our analyses on these 21 studies. Figure 1 provides a PRISMA flow diagram showing search results.

Due to the breadth of the topic and the variety of variables included in this systematic review, the subject matter was organized according to the categories of psychopathological symptoms of interest in the study (Tables 1 and 2) and a final mixed category (Table 3). Of 21 studies meeting the inclusion criteria, there were 2 studies where self-harming behaviours and thoughts was the outcome in TGNC adolescents (Table 1), 6 studies where suicide was the outcome (Table 2), and 13 studies where the outcome was committing suicide combined to self-injurious attitudes (Table 3).

**Table 1:** Extracted data from included studies on gender dysphoria/transgender status and self-harming ideation/self-injurious behaviours

References	Country	Population/sample	Sample size	Demographics	Symptoms	Analysis	Results
Arcelus et al. [48]	U.K	Transgender adolescents (grouped in No NSSI; Lifetime NSSI)	268	(< 25 year old) x̄ = 19.9 ± 2.17 Individuals assigned female at birth (n = 121) Individuals assigned male at birth (n = 136)	NSSI	Descriptive statistics χ^2^ (sex at birth)	N = 144 (53.7%) reporting no lifetime NSSI N = 124 (46.3%) reporting lifetime NSSI **Lifetime NSSI *vs.* Lifetime Absence of NSSI** No significant difference in gender assigned at birth (*χ*^2^ = 3.53, *p* < .06), with individuals assigned female at birth (n = 64) showing a trend toward more NSSI than individuals assigned male at birth (n = 56) **Current *vs.* non-current NSSI** Significantly more individuals assigned female at birth (n = 45) engaging in NSSI than individuals assigned male at birth (n = 30) (χ^2^ = 7.09, *p* < .01)
Butler et al. [49]	U.K	Transgender adolescents, Other, Cisgender adolescents	8440 Male = 3625 Female = 4361 Other = 227 Transgender = 55	13–17–year old	Self-harm prevalence and ideation	χ^2^ Descriptive statistics	Significant difference between groups about rate of Self-harm thoughts χ^2^[12] = 805.73, *p* < .0005, Cramer’s *V* = 0.20) None of the time Trans = 17 (30.9%) Other = 116 (51.1%) Male = 2800 (77.2%) Female = 2524 (57.9%) All of the time Trans = 19 (34.5%) Other = 23 (10.1%) Male = 26 (0.7%) Female = 100 (2.3%)

Demographics regarded Age, x̄, in years, ± SD and Sex assigned at birth; *NSSI* non-suicidal self-injury, *x̄* mean, ± *SD* standard deviation

**Table 2:** Extracted data from included studies on gender dysphoria and suicidality (including suicidal ideation, suicide attempts and behaviours, suicide risk)

References	Country	Population/sample	Sample size	Demogr	Symptoms	Analysis	Results
Toomey et al. [39]	USA	Adolescents referred for GD (including AFAB, AMAB, nonbinary, questioning); Cisgender adolescents	120 617 AMAB n = 2020 (0.2%) AFAB n = 175 (0.1%) Nonbinary n = 344 (0.3%) Questioning n = 1052(0.9%) Female n = 60,973(50.6%) Male n = 57,871(48%)	11–19 year old x̄ = 14.7 ± 1.8	Suicide attempts, suicide behaviour	Descriptive statistics χ^2^ χ^2^ in R with Post hoc* comparisons* *Bivariate level* Logistic regressions	**Rates of attempted suicide** AFAB (50.8%) Nonbinary (41.8%) AMAB (29.9%) Questioning (27.9%) Female (17.6%) Male (9.8%) **Association between suicide behaviour and gender identity** (*χ*^2^*df* = 5] = 2279.2; *p* < 0.001)) **Significant difference between groups except for AMAB and nonbinary** (*χ*^2^[df = 1] = 0.08; *p* = 0.78), **and AFAB and nonbinary** (*χ*^2^[df = 1] = 3.66; *p* = 0.08) **Rates of suicide behaviours**: AFAB: (50.9% [95% CI = 45.51 to 58.17]) Nonbinary: (41.8% [95% CI = 36.57 to 47.22]) AMAB: (30.0% [95% CI = 24.06 to 36.59]) Questioning adolescents: (27.9% [95% CI = 25.27 to 30.69]) Female: (17.6% [95% CI = 17.31 to 17.91]) Male: (9.8% [95% CI = 9.59 to 10.08]) OR (95% CI) for: Female 1.37% [1.34 to 1.39] AMAB 1.27% [1.04 to 1.54] AFAB 1.84% [1.52 to 2.24] Nonbinary 1.38 [1.20 to 1.58] Questioning 1.40 [1.28 to 1.52]
Heino et al. [29]	Finland	Adolescents reporting transgender identity (0.2%); other/nonbinary identity (2.9%); cisgender identity (96.9%)	1425	15–16 year old x̄ = 15.59 ± 0.41	Severe suicidal ideation	Descriptive statistics Logistic regression for associations between transgender identity and suicidal ideation	Transgender identity reporting severe suicidal ideation significantly more commonly than cisgender identity (14.3 *vs.* 1.6%, *p* < 0.001) **OR (95% CI):** Model 1 (controlling for age, sex, honesty of responding): Significant association between transgender status and severe suicide ideation Significantly higher severe suicidal ideation in people identifying themselves as transgender (OR [95% CI] = 10.8[4.0 to 28.9], *p* < 0.001) Model 2 (controlling for socioeconomic factors): Significant association between transgender identity and suicidal ideation = (OR [95% CI] = 9.9 [3.0 to 32.1], *p* < 0.001) Model 3 (controlling for depression): Significant association = (OR [95% CI] = 6.3 [1.6 to 24.9], *p* = 0.009); Model 4 (controlling for peer rejection and victimization): Significant association = (OR [95% CI] = 5.3 [1.3 to 22.1], *p* = 0.024)
Fisher et al. [17]	Italy	Age-matched GDs and NTs control group	92 GDs = 46 NRs = 46	GDs x̄ = 16 ± 1.49 NRs x̄ = 16.59 ± 1.11	Suicide risk	(ANCOVA)	Significant difference between GDs and NRs in: “Attraction to death”: 2.98 ± 0.57 *vs.* 2.17 ± 0.58; F = 46.22, *p* < 0.0001 “Repulsion by life” scales: 3.04 ± 0.46 *vs*. 2.08 ± 0.56, F = 78.5, *p* < 0.0001 “Attraction to life” scales: 3.32 ± 0.55 *vs*. 4.05 ± 0.49, F = 44.14, *p* < 0.0001 Significant difference between GDs and NRs on the “Internalizing scale” (depression/anxiety): 62.43 ± 11.18 *vs.* 53.57 ± 11.64, F = 12.53 *p* = 0.001
Yüksel et al. [51]	Turkey	Transgender adolescents	141 diagnosed with transsexuality (DSM-IV)	x̄ = 27.5 ± 7.16 AFAB = 99 AMAB = 42	Suicide attempts, suicidal ideation	Descriptive statistics χ^2^ for gender differences	**Suicidal rates (%)** Current suicidal thoughts 9.2% Lifetime suicidal thought (n = 78) 55.3% Suicide attempts (n = 42) 29.8% Before 21 yr of age:76.7% After 21 yr of age: 23.3% **Not significant**: Current suicidal thoughts AFAB-AMAB,* χ*^2^ = 0.515 Lifetime suicidal thoughts AFAB-AMAB, *χ*^2^ = 0.515 Suicide attempts AFAB-AMAB, *χ*^2^ = 0.039
de Graaf et al. [24]	Canada, Netherlands, U.K	Adolescents referred for GD	2771 Toronto (n = 260) Amsterdam (n = 266) London (n = 2245)	**Total sample** x̄ = 15.99 ± 1.20 **Toronto** x̄ = 16.66 ± 1.75 Male N (%) 129 (49.6%) Female N (%) 131 (50.4%) **Amsterdam** x̄ = 15.91 ± 1.42 Male N (%) 123 ± 46.2 Female N (%) 143 ± 53.8 **London** x̄ = 15.93 ± 1.07 Male N (%) 685 (30.5%) Female N (%) 1560 (69.5%)	Suicidality	Comparative analysis Multiple linear regression analysis	Clinic-referred sample ranges for CBCL: Item 91 (suicidal ideation) 17.9%—34.9%; Item 18 (suicidal behaviour) 7.7%—31.1% Non-referred sample ranges: Item 91: 1.4%—2.7%; Item 18: 0.6%—1.8% Clinic-referred samples ranges for YSR: Item 91 14.4–31.2.%; Item 18: 7.5–24.3% Non-referred sample ranges: Item 91: 1.5%–7.7%; Item 18: 2.2–3.9% Birth assigned sex (predictor) β = 0.105 (Toronto *vs*. Amsterdam) Birth assigned sex β = 0.035 (Toronto *vs.* London) Birth assigned sex β = 0.026 (Amsterdam *vs.* London)
Alizadeh Mohajer et al. [6]	Iran	Individuals diagnosed with GD	21	x̄ = 19	Suicidal ideation	Descriptive statistics Linear regression analysis	Beck’s Scale for Suicide Ideation No. (%) Low Risk 4 (19.1%) High Risk 15 (71.4%) Very High Risk 2 (9.5%) Mean of total score for Beck’s Scale for Suicide Ideation: CI 95%, 11.6 [8.7 to 15.6]

*AFAB* individuals assigned female at birth whose experienced gender is male, *AMAB* individuals assigned male at birth whose experienced gender is female, Demogr., demographics, i.e., (Age) Mean age, SD; Sex assigned at birth, *df* degrees of freedom, *GD* gender dysphoria, *GDs* gender dysphoric adolescents, *NRs* non-referred adolescents, *OR* odds ratio, x̄, mean, ± (SD), standard deviation

**Table 3:** Data extracted from included studies on gender dysphoria/TGNC/TNB, and both suicidality and self-harm

References	Country	Population/sample	Sample size	Demogr	Symptoms	Analysis	Results
Mak et al. [50]	USA	TGD	6327	18–25 year old	Suicide attempts/self-inflicted injury rates	Multivariable Cox regression analyses	**Incidence of the first self-harm—HR (95% CI)** Overall cohort 3.25 [1.84 to 5.74] AFAB 3.76 (1.79 to 7.89) AMAB 2.57 (1.01 to 6.55) **Incidence of Recurrent Self-Harm- HR (95%CI)** Overall cohort 2.87 (1.65 to 4.97) AFAB TF cohort 3.57 (1.77 to 7.21) AMAB 2.13 (0.90 to 5.03)
Thoma et al. [25]	USA	Transgender (including gender-nonconforming, nonbinary, questioning gender identity) and cisgender adolescents	2020 (1148 TGAs) (872 CGAs)	14–18 year old Transgender x̄ = 16.0 ± 1.2 Cisgenders x̄ = 15.9 ± 1.1	Passive death wish; suicidal ideation; suicide plan; suicide attempt; NSSI	Descriptive statistics for suicidality outcome within gender identity groups (95%CI) Multivariate logistic regression Multivariate logistic regression adjusted models	**Suicidal ideation (range):** All transgender (84.8%) (82.7% to 86.9%) All cisgender (60.4%) (57.1% to 63.7%) Nonbinary assigned female (79.2%) (75.1% to 83.3%) Nonbinary assigned male (72.1%) (58.5% to 85.7%) Questioning (82.4%) (71.8% to 93.0%) **NSSI (range):** All transgender status (86.9%) (84.9% to 88.9%) All cisgender (59.1%) (55.8% to 62.4%) Nonbinary assigned female (87.6%) (84.2% to 91.0%) Nonbinary assigned male (76.7%) (63.9% to 89.5%) Questioning 76.5(%) (64.7% to 88.3%) 95% CI **All transgender status (range)** Passive death wish: 93.0% to 95.6% Suicidal ideation: 82.7% to 86.9% Planning attempt: 70.0% to 75.1% Suicide attempt: 47.4% to 53.2% NSSI: 84.9% to 88.9% **Non-binary assigned female (range)** Passive death wish: 89.6% to 95.0% Suicidal ideation: 58.5% to 85.7% Planning attempt: 64.9% to 74.3% Suicide attempt: 39.3% to 49.3% NSSI: 84.2% to 91.0% **Non-binary male (range)** Passive death wish: 75.5% to 96.5% Suicidal ideation: 58.5% to 85.7% Planning attempt: 33.7% to 63.9% Suicide attempt: 29.2% to 59.2% NSSI: 95% CI 63.9% to 89.5% **All cisgender (range)** Passive death wish: 74.2% to 79.8 8% Suicidal ideation: 57.1% to 63.7% Planning attempt: 46.5% to 53.1% Suicide attempt: 28.3% to 34.5% NSSI: 55.8% to 62.4% **Higher OR for TGA**s: Passive Death Wish (OR = 2.60) Suicidal Ideation (OR = 2.20) Planning attempt (OR = 1.82) Suicidal attempt (OR = 1.65) Attempt requiring medical care (OR = 2.01) NSSI (OR = 2.88)
Veale et al. [52]	Canada	TNB youth; Cisgender population	923	14–18 year old (n = 323) 19–25 year old (n = 600) 139 transgender girls/women (16.6%) 356 transgender boys/men (42.4%) 344 non-binary (41.0%), mostly females at birth (n = 283)	Self-harm; suicidal ideation; suicide attempts	*T*-test with Cohen’s d/χ^2^ for comparing transgender youth and general cisgender population mean scores ANOVA post hoc Tukey comparisons for mean scores: differences between subgroup	**Ages 14–18 years** Considering **suicide** (past year) BCAHS in school: 13.0% Trans youth entire sample: n = 199 65.2%; *χ*^2^_(df 1)_ = 472.56** (*p* < 0.01); Effect size (RR = 5.02) Times attempted (past year) BCAHS in school: x̄ ± SD = 0.11 ± 0.47 Trans youth entire sample: n = 199 x̄ ± SD = 0.65 ± 1.00; Student’s *t*_(198)_ = 7.62** (*p* < 0.01); Effect size (*d* = 1.15) At least one attempt (past year) BCAHS in school: 6.5% Trans youth entire sample: N = 199 36.1%; *χ*^2^_(d.f. 1)_ = 290.64** (*p* < 0.01); Effect size (RR = 5.55) **Self-harm** Number of times (past year) BCAHS in school: x̄ ± SD = 0.41 ± 1.03 Trans youth entire sample: n = 231 x̄ ± SD = 1.87(1.27); Student’s *t*_(230)_ = 17.47** (*p* < 0.01); Effect size (*d* = 1.42) At least once in the past year BCAHS in school: 16.5% Trans youth entire sample: n = 231 74.9%; *χ*^2^_(d.f. 1)_ = 540.93** (*p* < .01); Effect size (RR = 4.54) **Ages 19–25** Considered suicide (ever): CCHS (2012) 15.4%; TYHS (2014): n = 336 74.4%; *χ*^2^_(d.f. 1)_ = 893.37** (*p* < 0.01); Effect size (RR = 4.8) Attempted suicide (ever): CCHS 3.4%; TYHS: n = 156 37.7%; *χ*^2^_(d.f.1)_ = 552.30** (*p* < 0.01); Effect size (RR = 11.12) **14–18–years-old** General mental health *F*(2,234) = 0.54 Seriously considered suicide (past year): Trans boys/men n = 90 68.9%; Trans Girls/Women n = 20; 55.0%; Non-binary n = 93; 64.5%; *χ*^2^_(d.f. 2)_ = 1.47 Self-harm at least once in the past year Trans Boys/men n = 106 79.2% Trans Girls/Women n = 24; 50.0%; Non-binary n = 100 77.0%; *χ*^2^_(d.f. 2)_ = 9.28* (*p* < .05) **19–25 years** General mental health *F*_(2,407)_ = 4.71 (*p* < 0.01) Seriously considered suicide (ever) Trans Boys/men n = 139 76.8%; Trans Girls/Women n = 65; 66.2% Non-binary n = 97; 76.7%; *χ*^2^_(2)_ = 3.12 Self-harm at least once in the past year Trans Boys/men n = 150; 48.0%; Trans Girls/Women n = 77; 40.3%; Non-binary n = 150; 60.7%; *χ*^2^_(2)_ = 9.78** (*p* < 0.01)
Wang et al. [11]	China	TGNC (including nonbinary, questioning); Cisgender adolescents	12 108 2111 TGNC 9997 Cisgender	x̄ = 15.8 ± 1.0 AMAB = 6518 AFAB = 5590	Self-harm; suicidal ideation	Descriptive Statistics Linear mixed-model analysis Mixed-Effects Logistic Regression	x̄ (SD) for Self-harm and suicidal ideation: **Transgender** AMAB = 4.03 (6.44%); AFAB = 3.51 (5.47%) **Nonbinary** AMAB = 3.93 (6.69%); AFAB = 3.86(5.82) **Questioning** AMAB = 2.57(4.72%); AFAB = 3.17(5.12) **Cisgender** Boys 1.25 (3.60%); Girls 1.54 (3.79) TGNC reporting a significantly lower overall health (*t*_11.872_ = − 7.36; *p* < 0.001), higher suicide ideation (*t*_11.860_ = 12.22; *p* < 0.001), higher depression (*t*_11.830_ = 12.43; *p* < 0.001), anxiety symptoms (*t*_11.847_ = 11.47; *p* < 0.001) and higher sleep problems (*t*_11.683_ = 10.49; *p* < 0.001) than cisgender youth Youth AFAB reporting significantly lower level of overall health (*t*_11.866_ = − 7.91, *p* < 0.001), higher suicide ideation (*t*_11.866_ = 3.44* p* < 0.001), higher depression (*t*_11.827_ = 3.88, *p* < 0.001), higher anxiety symptoms (*t*_11.845_ = 8.71, *p* < 0.001) and sleep problems (*t*_11.676_ = 2.79, *p* < 0.005) than AMAB youth Compared with cisgender boys, significantly higher incidence (OR) of **Past suicide attempts** for: Transgender girls: OR, 4.35; 95% CI, 2.88 to 6.56 Transgender boys: OR, 2.92; 95% CI, 2.26 to 3.76 Nonbinary AMAB: OR, 3.94; 95% CI, 2.36 to 6.55 Nonbinary AFAB: OR, 3.06; 95% CI, 1.67 to 5,63 Questioning youth AMAB: OR, 2.61; 95% CI, 1.73 to 3.94 **Thoughts of self-harm** for: Transgender girls: OR, 3.06; 95% CI, 2.24 to 4.19 Transgender boys: OR, 4.06; 95% CI, 3.47 to 4.74 **Suicide plan in the past month** for: Transgender girls: OR, 4.44; 95% CI, 2.88 to 6.83 Transgender boys: OR, 2.66; 95% CI, 2.03 to 3.50 Nonbinary youth AMAB: OR, 5.36; 95% CI, 3.22 to 8.93 Nonbinary youth AFAB: OR, 4.06; 95% CI, 2.25 to 7.30 Questioning youth AFAB: OR, 2.36; 95% CI, 1.63 to 3.43 **Thoughts of suicide** for: Transgender girls: OR, 3.93;95% CI, 2.88 to 5.38 Transgender boys: OR, 3.71; 95% CI, 3.10 to 4.21 **Deliberate self-harm during the last month** for: Transgender girls: OR, 2.74; 95% CI, 1.93 to 3.91 Transgender boys: OR, 3.06; 95% CI, 2.57 to 3.66 Nonbinary AMAB: OR, 2.56; 95% CI, 1.66 to 3.94 Nonbinary AFAB: OR, 3.06; 95% CI, 1.95 to 4.81
Becerra-Culqui et al. [53]	USA	TGNC; cisgender youth	1082 Transfeminine Cohort = 427; 73% Reference males = 4206; 72% Reference females = 4204; 72‰) Transmasculine Cohort = 655; 88% Reference males = 6448; 88% Reference females = 6459; 88%	10–17 year old	Suicidal ideation; self-inflicted injuries	Logistic Regression	**Prevalence Ratios (RPs)**: Transfeminine subjects compared with reference males for suicidal ideation: 54 (95% CI 18 to 218); For self-inflicted injuries: 70 (95% CI 9.0 to 159). Transmasculine subjects compared with reference males for suicide ideation: 45 [95% CI 23 to 97) For self-inflicted injuries: 144 (95% CI 14 to 4338)
Bechard et al. [54]	Canada	Adolescents diagnosed with Gender Identity Disorder (GID) (DSM-IV-TR)	50	13–20 year old x̄ = 16.9 Individuals assigned male at birth = 17 Individuals assigned female at birth = 33	Self-harm, suicidal ideation, and suicide attempts	Descriptive statistics	n(%) Suicidal ideation = 31 (62%) Self-harm = 18 (36%) Suicide attempt(s) = 13 (26%)
Kozlowska et al. [21]	Australia	Children and Adolescents referred for GD; Healthy Cisgenders	79 (GD) 155 (CIS)	GDs: 8.42–15.92 year old x̄ = 12.84 ± 1.90 Median = 13.33 Biological males = 33 (41.8%) Biological females (XX) = 46 (58.2%) CIS: 8.33–15.97 yr old x̄ = 12.9	Suicidal ideation, self-harm, suicide attempts	Descriptive statistics *T-*test/χ^2^	Suicidal ideation (past or current): n = 33 (41.8%) History of self-harm: n = 39 (16.3%) Suicide attempts: n = 8 (10.1%) Depressive disorder: n = 49 (62.0%) Anxiety disorder: n = 50 (63.3%) Significantly higher (*t*_[61.79]_ = 11.946; *p* < 0.001) *Depression, Anxiety, and Stress Scale* total scores between youth with GD and cis-controls Significant difference between children with high (57/79; 72.2%) and low (22/79; 27.8%) levels of gender distress for: History of self-harm (*χ*^2^ = 11.86; *p* < 0.001); Suicidal ideation (*χ*^2^ = 6.98; *p* = 0.008)
Skagerberg et al. [22]	U.K	Adolescents referred for GD	125	x̄ = 13.56 ± 3.24 54.8% individuals assigned male at birth 45.2% individuals assigned female at birth	Suicide attempts; self-harming thoughts and behaviours	Descriptive statistics *χ*^2^	Self-harm (24%); Thoughts of self-harm (14%); Suicide attempts (10%) Males 16–18 yr of age (n = 20) Thoughts of self-harm (37%) Self-harm (26%) Females 12–15 yr of age n = 30) Thought of self-harm (10%) Self-harm (37%) Suicide attempts (17%) Actual self-harm more common in individuals assigned female at birth than individuals assigned male at birth (*χ*^2^[1] = 6.84, *p* < 0.01) No significant difference between individuals assigned female at birth and individuals assigned male at birth for suicide attempts (*χ*^2^[1] = 0.87, *p* > 0.05)
Hartig et al. [30]	Germany	TGNC adolescents referred for GD	343	11–18 year old x̄ = 15.47 ± 1.51 Male at birth (n = 56) x̄ = 15.69 ± 1.58 Female at birth (n = 287) x̄ = 15.42 ± 1.50	Risk of suicidal (suicidal ideation, suicide attempts) and non-suicidal self-harming thoughts and behaviours (STBs)	Descriptive statistics	CBCL parent-reported STBs for Item 18 (suicidal behaviour): O*ften* (9.5%); S*ometimes* (20%) Total = 29% Youth self-reported STBs: *often* (19%), *sometimes* (26%), Total = 45% CBCL parent-reported STBs for Item 91 (suicidal ideation): *Often* (2%); *Sometimes* (18%); Total = 20% Youth self-reported STB: O*ften* (13%), *Sometimes* (25%); Total = 38%
Peterson et al. [55]	USA	Adolescents diagnosed with GD	96 (AFAB = 54, AMAB = 31, Nonbinary/gender fluid = 15	12–22 year-old, x̄ = 17.1 ± 2.3	Suicide attempts; self-injurious behaviours	Descriptive statistics χ^2^	N (%) History of suicide attempt = 27 (30.3%) History of self-injurious behaviours = 40(41.8%) Depressive disorder (n = 36, 38%) Generalized anxiety disorder (n = 15, 16%) Significant difference for AFAB reporting more suicide attempt and self-harm compared with AMAB respectively: (*χ*^2^ = 9.38, *p* < 0.05; *χ*^2^ = 8.73, *p* < .05)
H. K. Mitchell et al. [56]	USA	TNB adolescents referred for GD	798,994	13–20 year old	Suicidality; self-harm; suicidality and self-harm combined	Multivariable Poisson regression models	**2016 cohort OR [95% CI]** Association with suicidality Unadjusted 6.19 [5.80 to 6.60]; Adjusted 3.80 [3.52 to 4.10] Association with self-harm Unadjusted 3.80 [3.13 to 4.61]; Adjusted 2.68 [2.17 to 3.30] Association with suicidality or self-harm Unadjusted 5.71 [5.30 to 6.06]; Adjusted 3.59 [3.35 to 3.85] **2019 cohort OR [95% CI]** Association with suicidality Unadjusted 4.71 [4.58 to 4.85]; Adjusted 3.23 [3.12 to 3.34] Association with self-harm Unadjusted 4.21 [3.85 to 4.61]; Adjusted 2.90 [2.62 to 3.21] Association with suicidality or self-harm Unadjusted 4.58 [4.46 to 4.69]; Adjusted 3.15 [3.06 to 3.25]
Karvonen et al. [57]	Finland	Gender-referred adolescents (GR) and adolescents mental health referred (MHR)	GR = 84 MHR = 293	**GR** x̄ = 16.2 ± 1.3 **MHR** x̄ = 15.6. ± 1.6 *p* = 0.002 More birth-assigned females in the GR group than in the MHR group (84.5% *vs*. 64.2%, *p* < 0.001)	Suicidal ideation and talk, suicide attempt; self-harming behaviours	ANOVA	**Suicidal ideation and talk** GR = 70.2%; MHR = 50.5%; ***p* = 0.001** OR [95% CI] Model 1 controlled for age and sex = 1.8 [1.0 to 3.1]; ***p* = 0.04** OR [95% CI] Model 2 controlled for age, sex, family and child welfare contacts = 2.8 [1.6 to 5.2]; ***p* = 0.001** **Suicide attempt** GR = 10.7%; MHR = 8.9%; *p* = 0.37 OR [95% CI] Model 1 controlled for age and sex = 1.0 [0.5 to 2.3]; *p* = 0.93 OR [95% CI] Model 2 controlled for age, sex, family and child welfare contacts = 1.1 [0.4 to 2.8]; *p* = 0.9 **Self-harming behaviours** GR = 61.4%; MHR = 39.2%; ***p* = < 0.001** R [95% CI] Model 1 controlled for age and sex = 1.9 [1.1 to 3.2]; ***p* = 0.02** OR [95% CI] Model 2 controlled for age, sex, family and child welfare contacts = 2.2 [1.2 to 4.0]; ***p* = 0.007** **Depression** GR = 67.9%; MHR = 59.0%; *p* = 0.09 OR [95% CI] Model 1 controlled for age and sex = 1.0 [0.6 to 1.7]; *p* = 0.97 OR [95% CI] Model 2 controlled for age, sex, family and child welfare contacts = 1.7 [0.9 to 3.0]; *p* = 0.1
Tordoff et al. [58]	USA	TNB adolescents	104, AMAB = 63; AFAB = 27; Nonbinary/gender fluid = 10; Questioning = 4	13–20 year old x̄ = 15.8 ± 1.6	Self-harm; suicidal thoughts	Descriptive statistics	No. (%) Self-harm or suicidal thoughts at baseline = 45 (43.2%) Depression at baseline 0–4 (minimal) = 14 (13.5%) 5–9 (mild) = 27 (26.0%) 10–14 (moderate) = 22 (21.2%) 15–19 (moderately severe) = 11 (10.6%) ≥ 20 (severe) = 26 (25.0%) Missing = 4 (3.8%) Anxiety at baseline 0–4 (minimal) = 20 (19.2%) 5–9 (mild) = 28 (26.9%) 10–14 (moderate) = 20 (19.2%) 15–19 (moderately severe) = 11 (10.6%) ≥ 20 (severe) = 32 (30.8%) Missing = 4 (3.8%)

*AFAB* individuals assigned female at birth whose experienced gender is male, *AMAB* individuals assigned male at birth whose experienced gender is female, Demogr., demographics, i.e., (Age) Mean age, SD; Sex assigned at birth, *GD* gender dysphoria, *NSSI* non-suicidal self-injury, *OR* odds ratio, *TGD* transgender and gender diverse individuals, *TGNC* transgender and gender non-conforming, *TNB* transgender and nonbinary, *x̄* mean, ± *(SD)* standard deviation

The 21 studies were mainly from the United States, the United Kingdom and Europe. Included studies were also conducted in China, Iran, Turkey, Canada and Australia. Studies were non-interventional and observational, with 17 being cross-sectional or retrospective and 4 longitudinal.

GD and non-suicidal self-harming ideation and behaviours were investigated by two studies [48, 49], GD and suicidality by six studies [6, 17, 24, 29, 39, 51], and GD and both suicidality and non-suicidal self-harm by 13 [11, 21, 22, 25, 30, 50, 52–58]; of these studies, four [11, 21, 55, 58] detected the presence of internalizing problems (depressive and anxiety disorders) in GD adolescents and young adults. Detailed results are provided in the Additional file 1.

### Summary results

Detailed results of each study are shown in Tables 1–3 and in the Additional file 1. We will here summarise results in GD and transgender populations regarding studies of (1) non-suicidal self-harm, (2) suicidal ideation and attempts, and (3) non-suicidal self-harm, suicidal ideation, and suicide attempts combined.

*- Non-suicidal self-harming* was explored in two studies [48, 49]; transgender adolescents showed higher tendency toward self-harm ideation than cisgender adolescents, while non-suicidal self-inflicted behaviours were more common in cisgender males and females than among transgender adolescents [49]. AFAB adolescents showed nominally more lifetime and current NSSI than AMAB, but this did not reach statistical significance [48] (Table 1).
*- Suicidal thinking and attempts only* were examined in six studies [6, 17, 24, 29, 39, 51] and generally identified a high prevalence in GD/transgender populations of suicide behaviours and attempts, ranging from 14.3% of severe suicidal ideation in Heino et al. [29] to a cumulative “high risk” of 80.9% in Alizadeh Mohajer et al. [6]; however, studies used different assessment instruments, so it becomes difficult to draw conclusions as to the real extent of suicidality in our target population. Gender-diverse adolescents displayed high suicidal ideation (Table 2). Transgender/GD adolescents displayed more suicidal behaviour than cisgender adolescents, either males or females [39]. Suicidality did not differ between AMAB and AFAB transgender adolescents/younger adults [51].
*- Suicidality and non-suicidal self-harm combined* were explored in thirteen studies [11, 21, 22, 25, 30, 50, 52–58]. Both NSSI and suicidality were higher in transgender/GD youths than in cisgender participants. AMAB and AFAB showed higher NSSI and suicidality rates than cisgender boys and girls [11] (Table 3).

Additional considerations will be detailed further on.

## Discussion

The last decade has seen an increase in cases of GD in adolescents worldwide and our knowledge of the epidemiological and clinical features continues to evolve [59]. An adequate understanding of the phenomenon and any related symptoms is important for the early management and possible prevention of distress. Indeed, the literature has highlighted the existence of a high association of psychological and psychiatric symptoms in adolescents with GD.

Several studies used different methods to investigate whether transgender identity and clinical outcomes in the general adolescent population are related [6, 17, 21, 22, 24, 25, 29, 30, 39, 48–58]. The present review focused particularly on high-severity psychological symptoms in young people, such as self-harm and suicidal symptomatology. Indeed, the results of the studies underline a statistically significant correlation between youth TGNC—including gender dysphoric, non-binary and questioning adolescents—and prevalence of suicidal thinking and plans/attempts and self-harming thoughts and behaviours compared to cisgender populations [20, 29, 60–65]. Currently, there is a dearth of results from population-based samples, hence generalizing current findings is still very premature [59]. Despite progress and availability of resilience factors to face stigma and discrimination in some societies and social groups, there are considerable anti-LGBT attitudes in some countries and other social groups, ensuing in GD adolescents showing more mental symptoms and distress compared to cisgender peers [52, 66, 67]. Gender dysphoric adolescents show higher rates of depression leading to suicidal risk and engage in more self-injurious behaviours than their cisgender peers, confirming that a significant proportion of this population experience severe suicidal ideation and almost one third attempt suicide [4]. Other studies highlight that half of transgender youths are diagnosed with depression and anxiety disorders as well as poorer overall health and sleep quality [11, 66, 68]. Furthermore, puberty appears to exacerbate mental health problems in people with GD [30].

The main theoretical models, such as the gender minority stress model [69], identify potential risk factors among transgender individuals, link exposure to stigma, discrimination, and lack of social support. Previous research identified sexual minority status as a fundamental risk factor for own life-threatening behaviours [70]. In fact, adolescents diagnosed with GD experience victimization from their peers, negative parental reactions to their gender-nonconforming expression and identity, and family violence. These exogenous factors often lead transgender individuals to experience personal distress and isolation, which might elicit higher rates of own-life-threatening behaviours, such as suicidal attempts and ideation and self-harm thoughts than their heterosexual peers [70–73].

Overall, results of the studies included in this systematic review confirmed that, compared to cisgender adolescents, TGNC adolescents reported a significantly higher frequency of suicidal attempts, suicidal thoughts, making suicide plans, self-harm ideation and deliberately participating in self-harm acts. Higher depressive and anxiety symptoms and lower overall physical health were also positively associated with GD [11, 55, 57, 58, 74].

However, results were heterogeneous. Specifically, Wang and colleagues [11] indicated that among the gender minority groups transgender girls had the greater risk of planning and attempting suicide, transgender boys had the highest risk of performing deliberate self-harm, and questioning youth AFAB had the highest risk of suicidal ideation. Similar results were obtained in another study [24], with the risk ratios analysis highlighting the greater rate of suicidality among birth-assigned females. This pattern is consistent with many other studies showing that suicidality is more common among AFAB adolescents than it is among AMAB youth [75]. Some studies found possible gender differences between AFAB and AMAB and possible consequences for their mental health, suggesting that although AMAB might experience more stigmatization and preconceptions, AFAB youth seem to cope differently with distress [17, 25, 48]. Nevertheless, this outcome was different from Toomey and colleagues’ work [39], which found that transgender boys had a higher rate of attempted suicide than transgender girls.

At any rate, despite these within-group discrepancies, general findings emerging from quantitative studies provide evidence that a large proportion of adolescents referred for GD and other transgender youth, whether “AFAB” or “AMAB”, have a substantial co-occurring history of psychosocial and psychological vulnerability, causing a higher risk for suicidal ideation and life-threatening behaviours, such as self-harm thoughts and self-injurious gestures [70, 76].

Since society is becoming increasingly liquid according to Zygmunt Bauman [77], more cases of transgender states and GD are anticipated to occur; this will mean that we will have more of the general population at enhanced risk for self-harming acts, suicidal thinking, and suicidal behaviour. Under this perspective, it should be important to develop a comprehensive psychological assessment aimed at identifying people at risk of the above behaviours so to enforce preventive programmes [78–80].

For this reason, results provided by this systematic review may enhance the knowledge of health professionals about adolescents referred for GD. Furthermore, a better understanding of the mental health status of transgender youth and the associated risks could help to develop and provide effective interventions. The need for more knowledge and tools is also a key aspect of supporting each individual properly [30, 81]. Finally, increasing social awareness and scientific knowledge can also help target support programs for parents. Indeed, parents could benefit from interventions dedicated to understanding the impact of attitudes, behaviours and decisions, as well as assisting them in the therapeutic paths they take with their children with GD [70].

*Limitations*. This review contains heterogeneous data that could not be subjected to a meta-analysis. Heterogeneity regarded the instruments used to assess the populations included and the variables examined. To add to the high heterogeneity, the population under study belonged to multiple categories, such as cisgender males, cisgender females, individuals assigned female at birth whose experienced gender was male (so-called female-to-male transgender), individuals assigned male at birth whose experienced gender was female (so-called male-to-female transgender), and nonbinary. Often studies did not differentiate possible transgender from nonbinary identities. We attempted at focusing on GD only, but had to deal also with other populations as well, since the literature treats these populations as they were one and the same, which of course is not the case. Furthermore, a distinction between self-harm and suicide attempts was not always possible. Moreover, the social stigma laid upon gender diverse populations and current cultural trends may have directly or indirectly affected the writing of this review and its final results and conclusions.

## Conclusions

In conclusion, the overall findings emerging from this review provide evidence that a large proportion of adolescents with GD have a substantial concomitant history of psychosocial and psychological vulnerability, with a higher risk of suicidal ideation, life-threatening behaviour, and self-injurious thoughts or self-harm. Understanding the mental health status of transgender young people could help developing and providing effective clinical pathways and interventions. The relatively new issue of suicide in adolescent/young adult populations currently suffers from poor assessment standardization. There is a need for standardized assessment, culturally adapted research, and destigmatisation of this socially vulnerable population to address the issue of increased suicidal thinking and attempts.

### Supplementary Information

**Additional file 1. **Supplement to Marconi et al. (2023)

## Data Availability

N/A.
