# Supplementary material for: A systematic review on gender dysphoria in adolescents and young adults: focus on suicidal and self-harming ideation and behaviours
Source: Child Adolesc Psychiatry Ment Health. 2023 Sep 21;17:110. doi: 10.1186/s13034-023-00654-3 (PMC10515052; doi:10.1186/s13034-023-00654-3)
Supplement: Supplementary file 1 — Additional file 1. Supplement to Marconi et al. (2023) [file 13034_2023_654_MOESM1_ESM.docx]

Supplement for Marconi et al.

**Risk-of-Bias of the included studies.**

| **Arcelus et al. (2016)** | YES (2) | PARTIAL (1) | NO (0) | N/A |
| --- | --- | --- | --- | --- |
| 1 Question / objective sufficiently described? | 2 |  |  |  |
| 2 Study design evident and appropriate? | 2 |  |  |  |
| 3 Method of subject/comparison group selection or source of information/input variables described and appropriate? | 2 |  |  |  |
| 4 Subject (and comparison group, if applicable) characteristics sufficiently described? | 2 |  |  |  |
| 5 If interventional and random allocation was possible, was it described? |  |  |  | N/A |
| 6 If interventional and blinding of investigators was possible, was it reported? |  |  |  | N/A |
| 7 If interventional and blinding of subjects was possible, was it reported? |  |  |  | N/A |
| 8 Outcome and (if applicable) exposure measure(s) well defined and robust to measurement/misclassification bias? Means of assessment reported? |  | 1 |  |  |
| 9 Sample size appropriate? |  | 1 |  |  |
| 10 Analytic methods described/justified and appropriate? | 2 |  |  |  |
| 11 Some estimate of variance is reported for the main results? | 2 |  |  |  |
| 12 Controlled for confounding? | 2 |  |  |  |
| 13 Results reported in sufficient detail? | 2 |  |  |  |
| 14 Conclusions supported by the results? | 2 |  |  |  |
| **Butler et al. (2019)** | YES (2) | PARTIAL (1) | NO (0) | N/A |
| 1 Question / objective sufficiently described? | 2 |  |  |  |
| 2 Study design evident and appropriate? | 2 |  |  |  |
| 3 Method of subject/comparison group selection or source of information/input variables described and appropriate? | 2 |  |  |  |
| 4 Subject (and comparison group, if applicable) characteristics sufficiently described? | 2 |  |  |  |
| 5 If interventional and random allocation was possible, was it described? |  |  |  | N/A |
| 6 If interventional and blinding of investigators was possible, was it reported? |  |  |  | N/A |
| 7 If interventional and blinding of subjects was possible, was it reported? |  |  |  | N/A |
| 8 Outcome and (if applicable) exposure measure(s) well defined and robust to measurement/misclassification bias? Means of assessment reported? | 2 |  |  |  |
| 9 Sample size appropriate? | 2 |  |  |  |
| 10 Analytic methods described/justified and appropriate? | 2 |  |  |  |
| 11 Some estimate of variance is reported for the main results? | 2 |  |  |  |
| 12 Controlled for confounding? |  | 1 |  |  |
| 13 Results reported in sufficient detail? | 2 |  |  |  |
| 14 Conclusions supported by the results? | 2 |  |  |  |
| **Toomey et al. (2018)** | YES (2) | PARTIAL (1) | NO (0) | N/A |
| 1 Question / objective sufficiently described? | 2 |  |  |  |
| 2 Study design evident and appropriate? | 2 |  |  |  |
| 3 Method of subject/comparison group selection or source of information/input variables described and appropriate? | 2 |  |  |  |
| 4 Subject (and comparison group, if applicable) characteristics sufficiently described? | 2 |  |  |  |
| 5 If interventional and random allocation was possible, was it described? |  |  |  | N/A |
| 6 If interventional and blinding of investigators was possible, was it reported? |  |  |  | N/A |
| 7 If interventional and blinding of subjects was possible, was it reported? |  |  |  | N/A |
| 8 Outcome and (if applicable) exposure measure(s) well defined and robust to measurement/misclassification bias? Means of assessment reported? | 2 |  |  |  |
| 9 Sample size appropriate? | 2 |  |  |  |
| 10 Analytic methods described/justified and appropriate? | 2 |  |  |  |
| 11 Some estimate of variance is reported for the main results? | 2 |  |  |  |
| 12 Controlled for confounding? | 2 |  |  |  |
| 13 Results reported in sufficient detail? | 2 |  |  |  |
| 14 Conclusions supported by the results? | 2 |  |  |  |
| **Heino et al. (2021)** | YES (2) | PARTIAL (1) | NO (0) | N/A |
| 1 Question / objective sufficiently described? | 2 |  |  |  |
| 2 Study design evident and appropriate? | 2 |  |  |  |
| 3 Method of subject/comparison group selection or source of information/input variables described and appropriate? | 2 |  |  |  |
| 4 Subject (and comparison group, if applicable) characteristics sufficiently described? | 2 |  |  |  |
| 5 If interventional and random allocation was possible, was it described? |  |  |  | N/A |
| 6 If interventional and blinding of investigators was possible, was it reported? |  |  |  | N/A |
| 7 If interventional and blinding of subjects was possible, was it reported? |  |  |  | N/A |
| 8 Outcome and (if applicable) exposure measure(s) well defined and robust to measurement/misclassification bias? Means of assessment reported? | 2 |  |  |  |
| 9 Sample size appropriate? | 2 |  |  |  |
| 10 Analytic methods described/justified and appropriate? | 2 |  |  |  |
| 11 Some estimate of variance is reported for the main results? | 2 |  |  |  |
| 12 Controlled for confounding? | 2 |  |  |  |
| 13 Results reported in sufficient detail? | 2 |  |  |  |
| 14 Conclusions supported by the results? | 2 |  |  |  |
| **Fisher et al. (2017)** | YES (2) | PARTIAL (1) | NO (0) | N/A |
| 1 Question / objective sufficiently described? | 2 |  |  |  |
| 2 Study design evident and appropriate? | 2 |  |  |  |
| 3 Method of subject/comparison group selection or source of information/input variables described and appropriate? | 2 |  |  |  |
| 4 Subject (and comparison group, if applicable) characteristics sufficiently described? | 2 |  |  |  |
| 5 If interventional and random allocation was possible, was it described? |  |  |  | N/A |
| 6 If interventional and blinding of investigators was possible, was it reported? |  |  |  | N/A |
| 7 If interventional and blinding of subjects was possible, was it reported? |  |  |  | N/A |
| 8 Outcome and (if applicable) exposure measure(s) well defined and robust to measurement/misclassification bias? Means of assessment reported? | 2 |  |  |  |
| 9 Sample size appropriate? |  | 1 |  |  |
| 10 Analytic methods described/justified and appropriate? | 2 |  |  |  |
| 11 Some estimate of variance is reported for the main results? | 2 |  |  |  |
| 12 Controlled for confounding? | 2 |  |  |  |
| 13 Results reported in sufficient detail? | 2 |  |  |  |
| 14 Conclusions supported by the results? | 2 |  |  |  |
| **Yüksel et al. (2017)** | YES (2) | PARTIAL (1) | NO (0) | N/A |
| 1 Question / objective sufficiently described? | 2 |  |  |  |
| 2 Study design evident and appropriate? | 2 |  |  |  |
| 3 Method of subject/comparison group selection or source of information/input variables described and appropriate? | 2 |  |  |  |
| 4 Subject (and comparison group, if applicable) characteristics sufficiently described? | 2 |  |  |  |
| 5 If interventional and random allocation was possible, was it described? |  |  |  | N/A |
| 6 If interventional and blinding of investigators was possible, was it reported? |  |  |  | N/A |
| 7 If interventional and blinding of subjects was possible, was it reported? |  |  |  | N/A |
| 8 Outcome and (if applicable) exposure measure(s) well defined and robust to measurement/misclassification bias? Means of assessment reported? | 2 |  |  |  |
| 9 Sample size appropriate? |  | 1 |  |  |
| 10 Analytic methods described/justified and appropriate? | 2 |  |  |  |
| 11 Some estimate of variance is reported for the main results? | 2 |  |  |  |
| 12 Controlled for confounding? | 2 |  |  |  |
| 13 Results reported in sufficient detail? | 2 |  |  |  |
| 14 Conclusions supported by the results? | 2 |  |  |  |
| **de Graaf et al. (2022)** | YES (2) | PARTIAL (1) | NO (0) | N/A |
| 1 Question / objective sufficiently described? | 2 |  |  |  |
| 2 Study design evident and appropriate? | 2 |  |  |  |
| 3 Method of subject/comparison group selection or source of information/input variables described and appropriate? | 2 |  |  |  |
| 4 Subject (and comparison group, if applicable) characteristics sufficiently described? | 2 |  |  |  |
| 5 If interventional and random allocation was possible, was it described? |  |  |  | N/A |
| 6 If interventional and blinding of investigators was possible, was it reported? |  |  |  | N/A |
| 7 If interventional and blinding of subjects was possible, was it reported? |  |  |  | N/A |
| 8 Outcome and (if applicable) exposure measure(s) well defined and robust to measurement/misclassification bias? Means of assessment reported? | 2 |  |  |  |
| 9 Sample size appropriate? | 2 |  |  |  |
| 10 Analytic methods described/justified and appropriate? | 2 |  |  |  |
| 11 Some estimate of variance is reported for the main results? | 2 |  |  |  |
| 12 Controlled for confounding? | 2 |  |  |  |
| 13 Results reported in sufficient detail? | 2 |  |  |  |
| 14 Conclusions supported by the results? | 2 |  |  |  |
| **Alizadeh Mohajer et al. (2020)** | YES (2) | PARTIAL (1) | NO (0) | N/A |
| 1 Question / objective sufficiently described? | 2 |  |  |  |
| 2 Study design evident and appropriate? | 2 |  |  |  |
| 3 Method of subject/comparison group selection or source of information/input variables described and appropriate? |  | 1 |  |  |
| 4 Subject (and comparison group, if applicable) characteristics sufficiently described? | 2 |  |  |  |
| 5 If interventional and random allocation was possible, was it described? |  |  |  | N/A |
| 6 If interventional and blinding of investigators was possible, was it reported? |  |  |  | N/A |
| 7 If interventional and blinding of subjects was possible, was it reported? |  |  |  | N/A |
| 8 Outcome and (if applicable) exposure measure(s) well defined and robust to measurement/misclassification bias? Means of assessment reported? | 2 |  |  |  |
| 9 Sample size appropriate? |  | 1 |  |  |
| 10 Analytic methods described/justified and appropriate? | 2 |  |  |  |
| 11 Some estimate of variance is reported for the main results? |  | 1 |  |  |
| 12 Controlled for confounding? | 2 |  |  |  |
| 13 Results reported in sufficient detail? | 2 |  |  |  |
| 14 Conclusions supported by the results? | 2 |  |  |  |
| **Mak et al. (2020)** | YES (2) | PARTIAL (1) | NO (0) | N/A |
| 1 Question / objective sufficiently described? | 2 |  |  |  |
| 2 Study design evident and appropriate? | 2 |  |  |  |
| 3 Method of subject/comparison group selection or source of information/input variables described and appropriate? | 2 |  |  |  |
| 4 Subject (and comparison group, if applicable) characteristics sufficiently described? | 2 |  |  |  |
| 5 If interventional and random allocation was possible, was it described? |  |  |  | N/A |
| 6 If interventional and blinding of investigators was possible, was it reported? |  |  |  | N/A |
| 7 If interventional and blinding of subjects was possible, was it reported? |  |  |  | N/A |
| 8 Outcome and (if applicable) exposure measure(s) well defined and robust to measurement/misclassification bias? Means of assessment reported? |  | 1 |  |  |
| 9 Sample size appropriate? | 2 |  |  |  |
| 10 Analytic methods described/justified and appropriate? | 2 |  |  |  |
| 11 Some estimate of variance is reported for the main results? | 2 |  |  |  |
| 12 Controlled for confounding? | 2 |  |  |  |
| 13 Results reported in sufficient detail? | 2 |  |  |  |
| 14 Conclusions supported by the results? | 2 |  |  |  |
| **Thoma et al. (2019)** | YES (2) | PARTIAL (1) | NO (0) | N/A |
| 1 Question / objective sufficiently described? | 2 |  |  |  |
| 2 Study design evident and appropriate? | 2 |  |  |  |
| 3 Method of subject/comparison group selection or source of information/input variables described and appropriate? | 2 |  |  |  |
| 4 Subject (and comparison group, if applicable) characteristics sufficiently described? | 2 |  |  |  |
| 5 If interventional and random allocation was possible, was it described? |  |  |  | N/A |
| 6 If interventional and blinding of investigators was possible, was it reported? |  |  |  | N/A |
| 7 If interventional and blinding of subjects was possible, was it reported? |  |  |  | N/A |
| 8 Outcome and (if applicable) exposure measure(s) well defined and robust to measurement/misclassification bias? Means of assessment reported? | 2 |  |  |  |
| 9 Sample size appropriate? | 2 |  |  |  |
| 10 Analytic methods described/justified and appropriate? | 2 |  |  |  |
| 11 Some estimate of variance is reported for the main results? | 2 |  |  |  |
| 12 Controlled for confounding? | 2 |  |  |  |
| 13 Results reported in sufficient detail? | 2 |  |  |  |
| 14 Conclusions supported by the results? | 2 |  |  |  |
| **Veale et al. (2017)** | YES (2) | PARTIAL (1) | NO (0) | N/A |
| 1 Question / objective sufficiently described? | 2 |  |  |  |
| 2 Study design evident and appropriate? | 2 |  |  |  |
| 3 Method of subject/comparison group selection or source of information/input variables described and appropriate? | 2 |  |  |  |
| 4 Subject (and comparison group, if applicable) characteristics sufficiently described? | 2 |  |  |  |
| 5 If interventional and random allocation was possible, was it described? |  |  |  | N/A |
| 6 If interventional and blinding of investigators was possible, was it reported? |  |  |  | N/A |
| 7 If interventional and blinding of subjects was possible, was it reported? |  |  |  | N/A |
| 8 Outcome and (if applicable) exposure measure(s) well defined and robust to measurement/misclassification bias? Means of assessment reported? | 2 |  |  |  |
| 9 Sample size appropriate? | 2 |  |  |  |
| 10 Analytic methods described/justified and appropriate? | 2 |  |  |  |
| 11 Some estimate of variance is reported for the main results? | 2 |  |  |  |
| 12 Controlled for confounding? | 2 |  |  |  |
| 13 Results reported in sufficient detail? | 2 |  |  |  |
| 14 Conclusions supported by the results? | 2 |  |  |  |
| **Wang et al. (2020)** | YES (2) | PARTIAL (1) | NO (0) | N/A |
| 1 Question / objective sufficiently described? | 2 |  |  |  |
| 2 Study design evident and appropriate? | 2 |  |  |  |
| 3 Method of subject/comparison group selection or source of information/input variables described and appropriate? | 2 |  |  |  |
| 4 Subject (and comparison group, if applicable) characteristics sufficiently described? | 2 |  |  |  |
| 5 If interventional and random allocation was possible, was it described? |  |  |  | N/A |
| 6 If interventional and blinding of investigators was possible, was it reported? |  |  |  | N/A |
| 7 If interventional and blinding of subjects was possible, was it reported? |  |  |  | N/A |
| 8 Outcome and (if applicable) exposure measure(s) well defined and robust to measurement/misclassification bias? Means of assessment reported? | 2 |  |  |  |
| 9 Sample size appropriate? | 2 |  |  |  |
| 10 Analytic methods described/justified and appropriate? | 2 |  |  |  |
| 11 Some estimate of variance is reported for the main results? | 2 |  |  |  |
| 12 Controlled for confounding? | 2 |  |  |  |
| 13 Results reported in sufficient detail? | 2 |  |  |  |
| 14 Conclusions supported by the results? | 2 |  |  |  |
| **Becerra-Culqui et al. (2018)** | YES (2) | PARTIAL (1) | NO (0) | N/A |
| 1 Question / objective sufficiently described? | 2 |  |  |  |
| 2 Study design evident and appropriate? | 2 |  |  |  |
| 3 Method of subject/comparison group selection or source of information/input variables described and appropriate? | 2 |  |  |  |
| 4 Subject (and comparison group, if applicable) characteristics sufficiently described? | 2 |  |  |  |
| 5 If interventional and random allocation was possible, was it described? |  |  |  | N/A |
| 6 If interventional and blinding of investigators was possible, was it reported? |  |  |  | N/A |
| 7 If interventional and blinding of subjects was possible, was it reported? |  |  |  | N/A |
| 8 Outcome and (if applicable) exposure measure(s) well defined and robust to measurement/misclassification bias? Means of assessment reported? | 2 |  |  |  |
| 9 Sample size appropriate? | 2 |  |  |  |
| 10 Analytic methods described/justified and appropriate? | 2 |  |  |  |
| 11 Some estimate of variance is reported for the main results? | 2 |  |  |  |
| 12 Controlled for confounding? | 2 |  |  |  |
| 13 Results reported in sufficient detail? | 2 |  |  |  |
| 14 Conclusions supported by the results? | 2 |  |  |  |
| **Bechard et al. (2017)** | YES (2) | PARTIAL (1) | NO (0) | N/A |
| 1 Question / objective sufficiently described? | 2 |  |  |  |
| 2 Study design evident and appropriate? | 2 |  |  |  |
| 3 Method of subject/comparison group selection or source of information/input variables described and appropriate? | 2 |  |  |  |
| 4 Subject (and comparison group, if applicable) characteristics sufficiently described? | 2 |  |  |  |
| 5 If interventional and random allocation was possible, was it described? |  |  |  | N/A |
| 6 If interventional and blinding of investigators was possible, was it reported? |  |  |  | N/A |
| 7 If interventional and blinding of subjects was possible, was it reported? |  |  |  | N/A |
| 8 Outcome and (if applicable) exposure measure(s) well defined and robust to measurement/misclassification bias? Means of assessment reported? |  | 1 |  |  |
| 9 Sample size appropriate? |  | 1 |  |  |
| 10 Analytic methods described/justified and appropriate? |  | 1 |  |  |
| 11 Some estimate of variance is reported for the main results? |  | 1 |  |  |
| 12 Controlled for confounding? |  | 1 |  |  |
| 13 Results reported in sufficient detail? | 2 |  |  |  |
| 14 Conclusions supported by the results? | 2 |  |  |  |
| **Kozlowska et al. (2021)** | YES (2) | PARTIAL (1) | NO (0) | N/A |
| 1 Question / objective sufficiently described? | 2 |  |  |  |
| 2 Study design evident and appropriate? | 2 |  |  |  |
| 3 Method of subject/comparison group selection or source of information/input variables described and appropriate? | 2 |  |  |  |
| 4 Subject (and comparison group, if applicable) characteristics sufficiently described? | 2 |  |  |  |
| 5 If interventional and random allocation was possible, was it described? |  |  |  | N/A |
| 6 If interventional and blinding of investigators was possible, was it reported? |  |  |  | N/A |
| 7 If interventional and blinding of subjects was possible, was it reported? |  |  |  | N/A |
| 8 Outcome and (if applicable) exposure measure(s) well defined and robust to measurement/misclassification bias? Means of assessment reported? | 2 |  |  |  |
| 9 Sample size appropriate? |  | 1 |  |  |
| 10 Analytic methods described/justified and appropriate? | 2 |  |  |  |
| 11 Some estimate of variance is reported for the main results? | 2 |  |  |  |
| 12 Controlled for confounding? | 2 |  |  |  |
| 13 Results reported in sufficient detail? | 2 |  |  |  |
| 14 Conclusions supported by the results? | 2 |  |  |  |
| **Skagerberg et al. (2013)** | YES (2) | PARTIAL (1) | NO (0) | N/A |
| 1 Question / objective sufficiently described? | 2 |  |  |  |
| 2 Study design evident and appropriate? | 2 |  |  |  |
| 3 Method of subject/comparison group selection or source of information/input variables described and appropriate? | 2 |  |  |  |
| 4 Subject (and comparison group, if applicable) characteristics sufficiently described? | 2 |  |  |  |
| 5 If interventional and random allocation was possible, was it described? |  |  |  | N/A |
| 6 If interventional and blinding of investigators was possible, was it reported? |  |  |  | N/A |
| 7 If interventional and blinding of subjects was possible, was it reported? |  |  |  | N/A |
| 8 Outcome and (if applicable) exposure measure(s) well defined and robust to measurement/misclassification bias? Means of assessment reported? | 2 |  |  |  |
| 9 Sample size appropriate? |  | 1 |  |  |
| 10 Analytic methods described/justified and appropriate? | 2 |  |  |  |
| 11 Some estimate of variance is reported for the main results? | 2 |  |  |  |
| 12 Controlled for confounding? | 2 |  |  |  |
| 13 Results reported in sufficient detail? | 2 |  |  |  |
| 14 Conclusions supported by the results? | 2 |  |  |  |
| **Hartig et al. (2022)** | YES (2) | PARTIAL (1) | NO (0) | N/A |
| 1 Question / objective sufficiently described? | 2 |  |  |  |
| 2 Study design evident and appropriate? | 2 |  |  |  |
| 3 Method of subject/comparison group selection or source of information/input variables described and appropriate? | 2 |  |  |  |
| 4 Subject (and comparison group, if applicable) characteristics sufficiently described? | 2 |  |  |  |
| 5 If interventional and random allocation was possible, was it described? |  |  |  | N/A |
| 6 If interventional and blinding of investigators was possible, was it reported? |  |  |  | N/A |
| 7 If interventional and blinding of subjects was possible, was it reported? |  |  |  | N/A |
| 8 Outcome and (if applicable) exposure measure(s) well defined and robust to measurement/misclassification bias? Means of assessment reported? |  | 1 |  |  |
| 9 Sample size appropriate? |  | 1 |  |  |
| 10 Analytic methods described/justified and appropriate? |  | 1 |  |  |
| 11 Some estimate of variance is reported for the main results? |  |  | 0 |  |
| 12 Controlled for confounding? |  | 1 |  |  |
| 13 Results reported in sufficient detail? | 2 |  |  |  |
| 14 Conclusions supported by the results? | 2 |  |  |  |
| **Peterson et al. (2017)** | YES (2) | PARTIAL (1) | NO (0) | N/A |
| 1 Question / objective sufficiently described? | 2 |  |  |  |
| 2 Study design evident and appropriate? | 2 |  |  |  |
| 3 Method of subject/comparison group selection or source of information/input variables described and appropriate? | 2 |  |  |  |
| 4 Subject (and comparison group, if applicable) characteristics sufficiently described? | 2 |  |  |  |
| 5 If interventional and random allocation was possible, was it described? |  |  |  | N/A |
| 6 If interventional and blinding of investigators was possible, was it reported? |  |  |  | N/A |
| 7 If interventional and blinding of subjects was possible, was it reported? |  |  |  | N/A |
| 8 Outcome and (if applicable) exposure measure(s) well defined and robust to measurement/misclassification bias? Means of assessment reported? | 2 |  |  |  |
| 9 Sample size appropriate? |  | 1 |  |  |
| 10 Analytic methods described/justified and appropriate? | 2 |  |  |  |
| 11 Some estimate of variance is reported for the main results? | 2 |  |  |  |
| 12 Controlled for confounding? | 2 |  |  |  |
| 13 Results reported in sufficient detail? | 2 |  |  |  |
| 14 Conclusions supported by the results? | 2 |  |  |  |
| **Mitchell et al. (2022)** | YES (2) | PARTIAL (1) | NO (0) | N/A |
| 1 Question / objective sufficiently described? | 2 |  |  |  |
| 2 Study design evident and appropriate? | 2 |  |  |  |
| 3 Method of subject/comparison group selection or source of information/input variables described and appropriate? | 2 |  |  |  |
| 4 Subject (and comparison group, if applicable) characteristics sufficiently described? | 2 |  |  |  |
| 5 If interventional and random allocation was possible, was it described? |  |  |  | N/A |
| 6 If interventional and blinding of investigators was possible, was it reported? |  |  |  | N/A |
| 7 If interventional and blinding of subjects was possible, was it reported? |  |  |  | N/A |
| 8 Outcome and (if applicable) exposure measure(s) well defined and robust to measurement/misclassification bias? Means of assessment reported? | 2 |  |  |  |
| 9 Sample size appropriate? | 2 |  |  |  |
| 10 Analytic methods described/justified and appropriate? | 2 |  |  |  |
| 11 Some estimate of variance is reported for the main results? | 2 |  |  |  |
| 12 Controlled for confounding? | 2 |  |  |  |
| 13 Results reported in sufficient detail? | 2 |  |  |  |
| 14 Conclusions supported by the results? | 2 |  |  |  |
| **Karvonen et al. (2022)** | YES (2) | PARTIAL (1) | NO (0) | N/A |
| 1 Question / objective sufficiently described? | 2 |  |  |  |
| 2 Study design evident and appropriate? | 2 |  |  |  |
| 3 Method of subject/comparison group selection or source of information/input variables described and appropriate? | 2 |  |  |  |
| 4 Subject (and comparison group, if applicable) characteristics sufficiently described? | 2 |  |  |  |
| 5 If interventional and random allocation was possible, was it described? |  |  |  | N/A |
| 6 If interventional and blinding of investigators was possible, was it reported? |  |  |  | N/A |
| 7 If interventional and blinding of subjects was possible, was it reported? |  |  |  | N/A |
| 8 Outcome and (if applicable) exposure measure(s) well defined and robust to measurement/misclassification bias? Means of assessment reported? | 2 |  |  |  |
| 9 Sample size appropriate? |  | 1 |  |  |
| 10 Analytic methods described/justified and appropriate? | 2 |  |  |  |
| 11 Some estimate of variance is reported for the main results? | 2 |  |  |  |
| 12 Controlled for confounding? | 2 |  |  |  |
| 13 Results reported in sufficient detail? | 2 |  |  |  |
| 14 Conclusions supported by the results? | 2 |  |  |  |
| **Tordoff et al. (2022)** | YES (2) | PARTIAL (1) | NO (0) | N/A |
| 1 Question / objective sufficiently described? | 2 |  |  |  |
| 2 Study design evident and appropriate? | 2 |  |  |  |
| 3 Method of subject/comparison group selection or source of information/input variables described and appropriate? | 2 |  |  |  |
| 4 Subject (and comparison group, if applicable) characteristics sufficiently described? | 2 |  |  |  |
| 5 If interventional and random allocation was possible, was it described? | 2 |  |  |  |
| 6 If interventional and blinding of investigators was possible, was it reported? |  |  |  | N/A |
| 7 If interventional and blinding of subjects was possible, was it reported? |  |  |  | N/A |
| 8 Outcome and (if applicable) exposure measure(s) well defined and robust to measurement/misclassification bias? Means of assessment reported? | 2 |  |  | N/A |
| 9 Sample size appropriate? |  | 1 |  |  |
| 10 Analytic methods described/justified and appropriate? | 2 |  |  |  |
| 11 Some estimate of variance is reported for the main results? |  | 1 |  |  |
| 12 Controlled for confounding? | 2 |  |  |  |
| 13 Results reported in sufficient detail? | 2 |  |  |  |
| 14 Conclusions supported by the results? | 2 |  |  |  |

N/A, not applicable. From: Kmet, L. M., Lee, R. C., & Cook, L. S. (2004) Standard Quality Assessment Criteria for Evaluating Primary Research Papers from a Variety of Fields. HTA Initiative # 13. Edmonton: Alberta Heritage Foundation for Medical Research. ISBN: 1-896956-79-3.

**Detailed results**

*Gender Dysphoria and non-suicidal self-harming ideation and behaviours*

Two studies [41, 42] investigated whether adolescents and young adults diagnosed with gender dysphoria and gender diverse youth are more likely to engage in self-harming thoughts and non-suicidal self-injurious behaviours compared to their cisgender peers (age range 13-25 years old). Overall, Chi-square (*χ*^2^) tests revealed a significant difference between these groups with regard to self-harm ideation *χ*[12] = 805.73, *p* < .0005, Cramer’s V= 0.20) [42]. Specifically, trans and Other adolescents are shown to have a higher tendency toward self-harm ideation than cisgenders, with 34.5% of trans and 10% of Other reporting to think about self-harm all of the time, compared to 0.7% of cisgender males and 2.3% of cisgender females [42, 19, 43], with 34.5% of trans and 10% of Other reporting to think about self-harm all of the time, compared with 0.7% of cisgender males and 2.3% of cisgender females. Consistently, thinking about non-suicidal self-inflicted behaviours none of the time it appears to be more common among cisgender males (77.2%) and cisgender females (57.9%) than among trans (30.9%) and Other (51.1%) [42]. In contrast, in another study [41] a lifetime presence of NSSI was identified in 46.3% of patients - with 28.73% currently engaging in NSSI – while no lifetime NSSI was observed in more than half of the sample (53.7%).

Furthermore, a chi-square test confirmed a nearly significant difference in gender assigned at birth (*χ*²=3.53, *p*<0.06) (*χ*²=7.09, *p*<0.01), with a tendency towards individuals assigned female at birth (n=64) displaying more lifetime and current NSSI compared to individuals assigned male at birth (n=56) [41].

*Gender Dysphoria and suicidality*

Six studies [2,11,18,23,33,44] were identified examining the association between gender dysphoria and gender diverse identity and suicidality in adolescents and young adults. Overall, there was a significant positive association between gender dysphoria identity and suicide behaviours and attempts (*χ*^2^[(df)=5]=2279.2; *p*<0.001) [33], with transgender identifying adolescents reporting severe suicidal ideation significantly more commonly than cisgender ones (14.3 *vs*. 1.6%, *p*<0.001) [23]. In the gender diverse adolescents’ samples, 19.1% had low-risk suicide ideation, 71.4% had high risk suicide ideation and 9.5% very high-risk suicide ideation, with linear regression analysis (CI 95%) indicating the mean of total score for the *Beck’s Scale for Suicide Ideation* (Beck et al., 1988) [45] being 11.6% (8.7% to 15.6%) [2]. Higher scores were also identified from the *Child Behaviour Checklist* (CBCL) items concerning suicidality in the clinic-referred samples of gender dysphoric adolescents, ranging from 17.9% to 34.9% and from 7.7% to 31.1% for “suicidal ideation” and “suicidal behaviour” respectively, compared to those showed by non-referred sample (1.4% to 2.7%; 1.5% to 7.7%; 2.2% to 3.9%) [18]. Furthermore, in the same study analogous data were obtained from the *Youth Self-Report* (YSR) (14.4% to 31.2.%; and 7.5% to 24.3% for clinic-referred samples, and 1.5% to 7.7%; 2.2% to 3.9% for non-referred samples). Potential suicidal differences between non-referred adolescents control groups (NRs) and gender dysphoric adolescents (GDs) were found, with a univariate analysis of variance (ANCOVA) indicating GDs scoring higher for suicidal risk, specifically in the “Attraction to death” (2.98 ± 0.57 vs. 2.17 ± 0.58; *F* = 46.22, *p* < 0.0001), “Repulsion by life “(3.04 ± 0.46 vs. 2.08 ± 0.56, *F* = 78.5, *p* < 0.0001) scales and scoring lower in the “Attraction to life” (3.32 ± 0.55 vs. 4.05 ± 0.49, *F* = 44.14, *p* < 0.0001) scale [11], measured on the *Multi-Attitude Suicide Tendency Scale for Adolescents* [46]. A logistic regression also confirmed a significant association between transgender identity and severe suicidal ideation persisting even after controlling for possible risk factors of suicidality such as age, sex and honesty (OR [95% CI]=10.8% [4.0% to 28.9%], p<0.001), age, sex, honesty and sociodemographic factors (OR [95% CI]=9.9% [3.0% to 32.1%], p<0.001), age, sex, honesty of responding, socioeconomic factors and depression (OR [95 % CI]=6.3% [1.6% to 24.9%], p=0.009). However, when adding peer rejection and victimization factors, the association between suicidal ideation and transgender identity decreased (OR [95 % CI]=5.3% [1.3% to 22.1%], p=0.024) [23].

Overall, as for groups differences, descriptive statistics suggested a higher prevalence of individuals assigned female at birth, whose experience is not congruent with their assigned sex (AFAB) (50.8%), Nonbinary (41.8%), individuals assigned male at birth, whose experience is not congruent with their assigned sex (AMAB) (29.9%) and Questioning (27.9%) self-reporting greater rates of suicide behaviours and attempted suicide compared to cisgender females (17.6%) and males (9.8%) [33], with the overall suicidal rates among gender dysphoria adolescents being 55.3% for lifetime suicidal thoughts, 96.7% occurring before reaching 21 years of age [44]. More specifically, a significant difference among AMAB, AFAB, nonbinary, questioning and cisgenders was found, except for AMAB and questioning adolescents (χ^2^[df = 1] = 0.08; *p* = .78) and AFAB and nonbinary (χ^2^[df = 1] = 3.66; *P* = .08), with analysis at a bivariate level indicating AFAB showing the highest rates of suicide behaviours than all other groups, followed by nonbinary youth (41.8% [95% CI: 36.57% to 47.22%]), AMAB (30.0% [95% CI=24.06% to 36.59%]) and questioning adolescents (27.9% [95% CI=25.27% to 30.69%]). Substantially lower rates of suicidality were observed in female cisgenders (17.6% [95% CI=17.31% to 17.91%]) compared to overall transgender groups, although they displayed higher scores than their male peers (9.8% [95% CI=9.59% to 10.08%]) [33]. By contrast, another study [44] found no statistically significant differences between AMAB or AFAB groups of transsexuals regarding current suicidal thought (χ^2^ =0.515), lifetime suicidal thoughts (χ^2^=0.515) and suicide attempts (χ^2^=0.039).

*Gender dysphoria, suicidality and self-harm*

Thirteen [8,15-16,19,24,43,47-49,50-53] examined whether adolescents and young adults diagnosed with gender dysphoria show a higher tendency for committing suicide correlated to self-harming attitudes. Overall, linear mixed-model analysis suggested that TGNC adolescents reported significantly lower overall health (*t*_11.872_=−7.36; *p*<0.001) and higher suicide and self-harm ideation (*t*_11.860_=12.22; *p*<0.001) than cisgender youth [8]. Veale and colleagues [47] also compared the mean scores of Canadian transgender individuals with those of cisgender population-based studies, with *t*-tests and *χ*² indicating that transgender youth have a higher risk for reporting self-harm, suicidal ideation, and suicide attempts in both the 14-18 and 19-25 groups (risk ratios 3.8% to 16.1%). In Tordoff and colleagues’ study [53] 45 (43.2%) transgender and nonbinary (TNB) adolescents reported self-harm or suicidal thoughts at baseline, before receiving gender-affirming care. Moreover, compared to mental health referred adolescents (MHR), gender-referred ones (GR) showed significantly higher suicidal ideation and talk, suicide attempt and self-harming behaviours [52, 51] (see Table 3). Also, in a sample of 79 youths referred for gender dysphoria, Kozlowska and colleagues [15] found that 33 of them (41.8%) reported past or current suicidal ideation, 39 (16.3%), a history of self-harm, and 8 (10.1%) suicide attempts. History of suicide attempts (n=27, 30.3%) and history of self-injurious behaviours (n=40, 41.8%) also emerged in another AFAB, AMAB, and nonbinary gender fluid group [50]. Previous research revealed similar data [16], reporting self-harm (24%), thoughts of self-harm (14%), and suicide attempts (10%) in 125 transgender adolescents; they also displayed higher rates of self-reported suicidal behaviours [*Often* (19%), *Sometimes* (26%)] and non-suicidal self-harming thoughts and behaviours (STBs), [*Often* (13%), *Sometimes* (25%)], respectively, compared to what their parents’ had reported [*Often* (9.5%), *Sometimes* (20%)] [24]. Similar results were found for longitudinal data with multivariable Cox regression analysis, indicating a high rate of suicide attempts at the first follow-up (3.25%, 95% CI=1.84 to 5.74) and somewhat lower rates during subsequent follow-ups (2.87%, 95% CI, 1.65% to 4.97%) [43].

With regard to subgroups differences, an ANOVA with *post hoc* Tukey comparisons indicated that transgender boys/men and nonbinary of a 19-25 years of age group had higher rates of self-harm and [47] (See Table 3). A greater prevalence of suicidal ideation (84.8%; range 82.7% to 86-9%) and NSSI (86.9%; range 84.9% to 88.9%), respectively, was found for all transgenders (86.9%; range 84.9% to 88.9%) and particularly for nonbinary assigned female (79.2%; range 75.1% to 83.3%; 87.6%; range 84.2% to 91.0%), nonbinary assigned male (72.1%; range 58.5% to 85.7%; 76.7%; range 63.9% to 89.5%), and questioning (82.4%; range 71.8% to 93.0%; 76%; range 64.7% to 88.3%) identity groups compared to their cisgender peers (60.4%; range 57.1% to 63.7%; 59.1%; range 55.8% to 62.4%) [19]. AMAB and AFAB also confirmed higher general mean rates for self-harm and suicide ideation, 4.03%; SD=6.44 and 3.51%; SD=5.47, respectively, compared to cisgender boys (1.25%; SD=3.60) and cisgender girls (1.54%; SD=3.79) [8]; the prevalence of self-harm and suicidal ideation and attempts was 62%, 36% and 26%, respectively [49].

Further mixed-effects logistic regressions have confirmed that, compared with cisgender boys, the gender minority groups as well as cisgender girls had a significantly higher likelihood of reporting self-harm thoughts [cisgender girls: (OR), 1.48%; 95% CI, 1.31% to 1.66%; AFAB (OR), 3.06%; 95% CI, 2.24% to 4.19%; AMAB (OR), 4.06%; 95% CI, 3.47% to 4.74%], suicidal thoughts [cisgender girls: (OR), 1.42%; 95% CI, 1.26% to 1.60%; AFAB (OR), 3.93% ;95% CI, 2.88% to 5.38%; AMAB (OR), 3.71%; 95% CI, 3.10% to 4.21%] as well as actual deliberate self-harm during the last month [cisgender girls: (OR), 1.49%; 95% CI, 1.33% to 1.68%; AFAB (OR), 2.74%; 95% CI, 1.93% to 3.91%; AMAB (OR), 3.06%; 95% CI, 2.57% to 3.66%; Nonbinary AMAB (OR), 2.56%; 95% CI, 1.66% to 3.94%; Nonbinary AFAB (OR), 3.06%; 95% CI, 1.95% to 4.81%]. Compared to cisgender boys, a significantly higher prevalence of past suicide attempts has also emerged in all gender minority groups [AFAB (OR), 4.35%; 95% CI, 2.88% to 6.56%; AMAB (OR), 2.92%; 95% CI, 2.26% to 3.76%%; Nonbinary AMAB (OR), 3.94%; 95% CI, 2.36% to 6.55%; Nonbinary AFAB (OR), 3.06%; 95% CI, 1.67% to 5.63%; Questioning youth AMAB (OR), 2.61%; 95% CI, 1.73% to 3.94%; Questioning youth AFAB (OR), 1.93%; 95% CI, 1.33% to 2.81%]. All gender minority groups, except questioning AMAB, were significantly more likely to report a suicide plan in the past month [AFAB (OR), 4.44%; 95% CI, 2.88% to 6.83%; AMAB (OR), 2.66%; 95% CI, 2.03% to 3.50%; Nonbinary AMAB (OR), 5.36%; 95% CI, 3.22% to 8.93%; Nonbinary AFAB (OR), 4.06%; 95% CI, 2.25% to 7.30%; Questioning AFAB (OR), 2.36%; 95% CI, 1.63% to 3.43%] [8]. More specifically, higher ORs were observed in all transgender, nonbinary assigned female, and nonbinary assigned male groups, respectively, regarding passive death wish (95% CI 93.0% to 95.6%; 95% CI 89.6% to 95.0%; 95% CI 75.5-% to 96.5%), suicidal ideation (95% CI 82.7% to 86.9%; 95% CI 58.5% to 85.7%; 95% CI 58.5% to 85.7%), planning attempts (95% CI 47.4% to 53.2%; 95% CI 39.3% to 49.3%; 95% CI 46.5% to 53.1%) and NSSI (95% CI 84.9% to 88.9%; 95% CI 84.2% to 91.0%; 95% CI 55.8% to 62.4%) than all cisgenders (Passive death wish: 95% CI 74.2% to 79.8 8%; suicidal ideation: 95% CI 57.1% to 63.7%; planning attempt: 95% CI 46.5% to 53.1%; suicide attempt: 95% CI 28.3% to 34.5%; NSSI: 95% CI 55.8% to 62.4%) [19]. The same authors applied adjusted multivariate logistic regression models, confirming higher ORs for all transgenders (passive death wish, 2.60; suicidal ideation, 2.20; planning attempt, 1.82; suicidal attempt, 1.65; attempt requiring medical care, 2.01; and NSSI, 2.88).

Concerning gender differences, *χ*^2^ showed a significant difference for AFAB reporting more suicide attempts (*χ*^2^=9.38, *p<0*.05) and self-harm (*χ*^2^=8.73, *p<0*.05) compared to AMAB [50], as well as actual self-harm being more common in individuals assigned female at birth than individuals assigned male at birth (*χ*^2^[1]=6.84, *p*<0.01). In contrast, another study found no significant differences between individuals assigned female at birth and individuals assigned male at birth for attempting suicide (*χ*^2^[1]=0.87, *p*>0.05) [16].

*Internalizing problems and general mental health in adolescents and young adults with Gender Dysphoria*

Four studies [8,15,50,53] included in this systematic review also detected the presence of internalizing problems – i.e., depressive and anxiety disorders – in gender dysphoric adolescents and young adults. For this reason, we inserted a separate paragraph dealing with these issues.

Overall, adolescents presenting (*t*_[61.79]_=11.946; *p*<0.001) with gender dysphoria scored significantly higher (*t*_[61.79]_=11.946; *p*<0.001) on total Depression, Anxiety, and Stress Scale (DASS) than healthy cis-controls. Three studies [15, 53, 50] using descriptive statistics found depression and anxiety disorders in children and adolescents with gender dysphoria, transgender, and nonbinary adolescents (Table 3). Concerning differences in gender assignment at birth, further linear mixed-model analysis also indicated that AFAB youths report significantly higher depression rates (*t*_[11.827]_=3.88, *p*<0.001), higher anxiety symptoms (*t*_[11 845]_=8.71, *p*<.001), and sleep problems (*t*_[11 676]_=2.79, *p*=0.005) than AMAB youth [8].


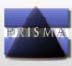
**PRISMA 2020 Checklist**

| **Section and Topic** | **Item #** | **Checklist item** | **Location where item is reported** |
| --- | --- | --- | --- |
| **TITLE** | | |  |
| Title | 1 | Identify the report as a systematic review. | 1 |
| **ABSTRACT** | | |  |
| Abstract | 2 | See the PRISMA 2020 for Abstracts checklist. | 2 |
| **INTRODUCTION** | | |  |
| Rationale | 3 | Describe the rationale for the review in the context of existing knowledge. | 3-4 |
| Objectives | 4 | Provide an explicit statement of the objective(s) or question(s) the review addresses. | 4 |
| **METHODS** | | |  |
| Eligibility criteria | 5 | Specify the inclusion and exclusion criteria for the review and how studies were grouped for the syntheses. | 4 |
| Information sources | 6 | Specify all databases, registers, websites, organisations, reference lists and other sources searched or consulted to identify studies. Specify the date when each source was last searched or consulted. | 4 |
| Search strategy | 7 | Present the full search strategies for all databases, registers and websites, including any filters and limits used. | 4 |
| Selection process | 8 | Specify the methods used to decide whether a study met the inclusion criteria of the review, including how many reviewers screened each record and each report retrieved, whether they worked independently, and if applicable, details of automation tools used in the process. | 4 |
| Data collection process | 9 | Specify the methods used to collect data from reports, including how many reviewers collected data from each report, whether they worked independently, any processes for obtaining or confirming data from study investigators, and if applicable, details of automation tools used in the process. | 4 |
| Data items | 10a | List and define all outcomes for which data were sought. Specify whether all results that were compatible with each outcome domain in each study were sought (e.g. for all measures, time points, analyses), and if not, the methods used to decide which results to collect. | 4 |
|  | 10b | List and define all other variables for which data were sought (e.g. participant and intervention characteristics, funding sources). Describe any assumptions made about any missing or unclear information. | 4-5 |
| Study risk of bias assessment | 11 | Specify the methods used to assess risk of bias in the included studies, including details of the tool(s) used, how many reviewers assessed each study and whether they worked independently, and if applicable, details of automation tools used in the process. | 4-5 |
| Effect measures | 12 | Specify for each outcome the effect measure(s) (e.g. risk ratio, mean difference) used in the synthesis or presentation of results. | 4-5 |
| Synthesis methods | 13a | Describe the processes used to decide which studies were eligible for each synthesis (e.g. tabulating the study intervention characteristics and comparing against the planned groups for each synthesis (item #5)). | N/A |
|  | 13b | Describe any methods required to prepare the data for presentation or synthesis, such as handling of missing summary statistics, or data conversions. | N/A |
|  | 13c | Describe any methods used to tabulate or visually display results of individual studies and syntheses. | 4,6,7-33 |
|  | 13d | Describe any methods used to synthesize results and provide a rationale for the choice(s). If meta-analysis was performed, describe the model(s), method(s) to identify the presence and extent of statistical heterogeneity, and software package(s) used. | N/A |
|  | 13e | Describe any methods used to explore possible causes of heterogeneity among study results (e.g. subgroup analysis, meta-regression). | N/A |
|  | 13f | Describe any sensitivity analyses conducted to assess robustness of the synthesized results. | N/A |
| Reporting bias assessment | 14 | Describe any methods used to assess risk of bias due to missing results in a synthesis (arising from reporting biases). | 4-5, Suppl. |
| Certainty assessment | 15 | Describe any methods used to assess certainty (or confidence) in the body of evidence for an outcome. | N/A |
| **RESULTS** | | |  |
| Study selection | 16a | Describe the results of the search and selection process, from the number of records identified in the search to the number of studies included in the review, ideally using a flow diagram. | 5-19 |
|  | 16b | Cite studies that might appear to meet the inclusion criteria, but which were excluded, and explain why they were excluded. | N/A |
| Study characteristics | 17 | Cite each included study and present its characteristics. | 12-19 |
| Risk of bias in studies | 18 | Present assessments of risk of bias for each included study. | Suppl |
| Results of individual studies | 19 | For all outcomes, present, for each study: (a) summary statistics for each group (where appropriate) and (b) an effect estimate and its precision (e.g. confidence/credible interval), ideally using structured tables or plots. | N/A |
| Results of syntheses | 20a | For each synthesis, briefly summarise the characteristics and risk of bias among contributing studies. | Suppl |
|  | 20b | Present results of all statistical syntheses conducted. If meta-analysis was done, present for each the summary estimate and its precision (e.g. confidence/credible interval) and measures of statistical heterogeneity. If comparing groups, describe the direction of the effect. | N/A |
|  | 20c | Present results of all investigations of possible causes of heterogeneity among study results. | 5-19 |
|  | 20d | Present results of all sensitivity analyses conducted to assess the robustness of the synthesized results. | 5-19 |
| Reporting biases | 21 | Present assessments of risk of bias due to missing results (arising from reporting biases) for each synthesis assessed. | Suppl |
| Certainty of evidence | 22 | Present assessments of certainty (or confidence) in the body of evidence for each outcome assessed. | N/A |
| **DISCUSSION** | | |  |
| Discussion | 23a | Provide a general interpretation of the results in the context of other evidence. | 20-22 |
|  | 23b | Discuss any limitations of the evidence included in the review. | 21 |
|  | 23c | Discuss any limitations of the review processes used. | 21 |
|  | 23d | Discuss implications of the results for practice, policy, and future research. | 20-22 |
| **OTHER INFORMATION** | | |  |
| Registration and protocol | 24a | Provide registration information for the review, including register name and registration number, or state that the review was not registered. | 20 |
|  | 24b | Indicate where the review protocol can be accessed, or state that a protocol was not prepared. | N/A |
|  | 24c | Describe and explain any amendments to information provided at registration or in the protocol. | N/A |
| Support | 25 | Describe sources of financial or non-financial support for the review, and the role of the funders or sponsors in the review. | 20 |
| Competing interests | 26 | Declare any competing interests of review authors. | 20 |
| Availability of data, code and other materials | 27 | Report which of the following are publicly available and where they can be found: template data collection forms; data extracted from included studies; data used for all analyses; analytic code; any other materials used in the review. | N/A, 20 |

*From:*  Page MJ, McKenzie JE, Bossuyt PM, Boutron I, Hoffmann TC, Mulrow CD, et al. The PRISMA 2020 statement: an updated guideline for reporting systematic reviews. BMJ 2021;372:n71. doi: 10.1136/bmj.n71

For more information, visit: <http://www.prisma-statement.org/>
